# Finding a way forward with the community: qualitative inquiry in the generalized HIV epidemic in Mizoram, India

**DOI:** 10.3389/fpubh.2023.1217628

**Published:** 2023-07-24

**Authors:** Amrita Rao, Megha Mamulwar, Samiran Panda, Henry Zodinliana Pachuau, H. Vanlalvenzuali, Lalruatsanga, Tarun Roy, Nunui Lalnuntlangi

**Affiliations:** ^1^Indian Council of Medical Research-National AIDS Research Institute (ICMR-NARI), Pune, Maharashtra, India; ^2^Indian Council of Medical Research, New Delhi, India; ^3^Department of Social Work, Mizoram University, Aizawl, Mizoram, India; ^4^ICMR-NARI Sustained & Timely AIDS Response: A Community Engagement in Mizoram (STAR) Project, Pune, Maharashtra, India

**Keywords:** Mizoram, HIV, generalized epidemic, restorative community building, community consultations

## Abstract

**Introduction:**

Mizoram, the northeastern State of India bordering Myanmar, is presently witnessing a burgeoning generalized HIV epidemic along with the highest State-level HIV prevalence among female sex workers (FSWs, 24.7%) and people who inject drugs (PWID,19.8%) in the country. The present study was conducted against such background to understand the current situation of HIV prevention and care services in Mizoram, and capture community voices as well as concerns so that the way forward could be informed appropriately.

**Methods:**

The study period was October through December 2020 (in the midst of COVID-restrictions) in the districts of Aizawl, Mamit, Kolasib, Lunglei, and Champhai where HIV prevalence crossed 1% among ante-natal clinic (ANC) attending women. Contrastingly, the national average HIV prevalence among ANC attendees is 0.24%, which formed the basis for selecting the aforementioned five high burden districts for this current inquiry. In-depth-interviews were conducted with community members and youth leaders, vulnerable and general population groups as well as HIV-program officials. Interviews were recorded, transcribed, translated and later coded for analysis following thematic approach.

**Results:**

The emerging issues were grouped in three thematic layers; (1) HIV vulnerability, (2) challenges pertaining to prevention and care services, and (3) program elements and future roadmap. Discrimination at the community level, unfriendly behavior of some of the HIV-service staff, occasional interruption in supplies of anti-retroviral treatment and sterile syringes and needles were voiced as deterrents to accessing HIV prevention and care services by the participants. Community engagement, based on restorative approach rather than retribution and ensuring enhanced performance of the district AIDS program control units emerged as necessary programmatic elements.

**Conclusion:**

This inquiry highlighted macro-social and structural forces contributing to stigma and discrimination toward people at risk of HIV. It is urgent that HIV-services are re-aligned through de-centralized district level innovations and creation of safer spaces at the physical, societal and familial level. These, even during the time of stress such as a pandemic, would help health services to remain resilient. HIV outreach, sensitization of the community leaders and health-care professionals through strategic communication and ownership of the communities in these endeavors appeared paramount.

## 1. Introduction

AIDS was first described in the year 1981 in the United States of America among men having sex with men and injecting drug users. Subsequently, it was described from other countries and was a cause of concern. The World Health Organization (WHO) declared AIDS as a global public health problem in 1986. In India, initially a task force was set up in the year 1985 by the Indian Council of Medical Research (ICMR), which was followed by a sero-survey in 1986 (1). The focus of this survey was on ‘most at risk population’ groups such as female sex workers (FSW), men having sex with men (MSM), transgender community (TG) and people who inject drugs (PWID), which revealed the first few cases among FSWs from Mumbai and Chennai in April 1986. Later, ante-natal clinic (ANC) attending women became part of the regular surveillance as surrogate for general population. During the years 1986 to 1991 the National AIDS Program worked in tandem with the ICMR to address issues around the AIDS pandemic (1) in the country.

In 1992, with the establishment of the National AIDS Control Organization (NACO), the National AIDS Control Program (NACP) institutionalized the beginning of comprehensive responses to HIV/AIDS epidemic in the country. Since then, the NACP has evolved through five phases; the first being executed during 1992-1999 and the ongoing one being the fifth (2021-2026) (2). Getting driven by in-country data has been the major strength of NACP. The NACO serves as the apex body managing HIV prevention, test and treatment program in India including maintenance of blood-safety, control of other sexually transmitted diseases as well as a range of harm reduction services (3). This works through the State AIDS Control Societies (SACS) and decentralized through the District AIDS Program Control Unit (DAPCU) as the grass root program implementation unit (4). The SACS in turn operate through collaboration with various medical colleges, hospitals, and healthcare facilities, research organizations, and community based or non-government organizations (CBOs/NGOs) in their respective States. Noticeably, the test and treat policy for management of HIV was rolled out in the year 2017 (5). The impact of these national AIDS responses in India has been significant. The estimated number of people living with HIV (PLHIV) in the country now, is about 23,31,476, with an HIV prevalence in adult general population of 0.21% and 84% of the PLHIV currently being on anti-retroviral treatment (ART) (6).

**Figure 1:**
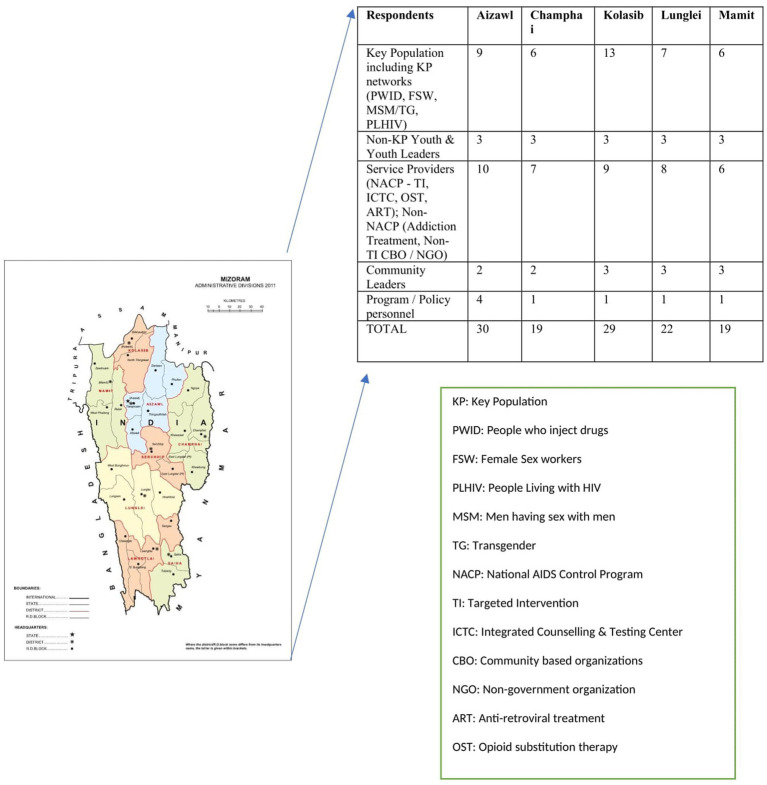
Map with respondent matrix.

The annual new HIV infections in India declined by 78% between 1997 and 2010 and about 46% between 2010 and 2021 (6, 7). AIDS deaths also decreased considerably with countrywide scaling up of ART However, such country averages do not reflect State-level heterogeneity and the achievements in terms of success in halting and reversing the HIV epidemic across the States have been disparate. The case in consideration is the rise in estimated annual new HIV infections in some of the north-eastern States such as Tripura, Arunachal Pradesh, Meghalaya, Assam, Sikkim, and Mizoram, and the union territory of Dadra and Nagar Haveli (7). Mizoram, is one of the north-eastern states that harbors 1.097 million population (8), with HIV prevalence in the age group 15–49 year at 2.7% (6). Additionally, while the national HIV prevalence among ANC attending women was recorded nationally at 0.22% (95% CI 0.21–0.24), it was five times higher at 1.13% (95% CI 0.79–1.47) in Mizoram (9). Five of the 11 districts of the State namely Aizawl, Kolasib, Mamit, Champhai, and Lunglei (Figure 1) had witnessed HIV prevalence in ANC attendees crossing 1%.

Importantly, a concerning level of vulnerability to HIV infection among the local youths in the north-eastern states of Manipur, Mizoram and Nagaland bordering Myanmar was recorded as early as in 1990s (10). Subsequent investigations identified various behavioral, socio economic, and geo-political factors including underdevelopment, poverty, proximity to golden triangle, and cross border trafficking of psychotropic substances, contributing to the burgeoning HIV epidemic (11) in this region. The HIV prevalence among FSW and PWID in the State, over the years, also reached concerning levels of 24.7 and 19.8%, respectively, (12).

The population in Mizoram mainly comprises of Christians (>90%) from different denominations – predominantly Presbyterian. Churches have been involved in many social issues including HIV/AIDS. It is also noteworthy that the Young Mizo Association (YMA), is the largest civil society organization (CSO) in the State with strong socio-cultural influence on the lives of people in different spheres, which encompasses health and development as well (13, 14). On the other hand, the hilly terrain of the State poses challenges to the delivery of effective health services (15).

Against this backdrop, we conducted the current qualitative investigation, which coincided with the time of COVID-19 in India. Our aim was to identify factors influencing the upsurge of HIV in Mizoram from the perspective of both key program officials and members of the different population groups in the community so that the ways forward for its containment could be identified.

## 2. Methodology

Study approval for the present inquiry was obtained from the Institutional Ethics Committee of the Indian Council of Medical Research-National AIDS Research Institute (ICMR-NARI) (NARI/EC Approval/20-21/409 dated 19th August 2020). Consultation of the research team members with the officials from NACO, Mizoram State AIDS Control Society (MSACS), Joint United Nations Program on HIV/AIDS (UNAIDS), Centre for Disease Control (CDC)-India office, field investigators on ground and key population groups helped in planning and finalization of the field operational plan for this qualitative investigation.

### 2.1. Gaining access

We adopted a purposive sampling strategy to identify participants from diverse lived experience of HIV vulnerability, program execution, organizing youths for social causes and community influencing activities to obtain wide ranging perspectives through qualitative inquiries. This strategy was deployed across the five study-districts where HIV prevalence among ANC attendees crossed 1%. We interacted with the members from Young Mizo Association, *Mizo Hmeichhe Insuihkhawm Pawl* (MHIP), Mizo Association for Women, *Mizo Zirlai Pawl* (MZP), Mizo Student’s Association and Women’s Anti-Drug Association (WADA). Potential respondents from the local communities such as people living with HIV (PLHIV), key population groups, service providers including peer educators (PE), religious leaders, policy and program officials, members of CBOs/CSOs/NGOs and local youths were contacted as described below. Three Community Based Organizations (CBOs) namely Mizoram Drug Users Forum (MDUF), Positive Women’s Network of Mizoram (PWNM) and Society for HIV/AIDS and Life Operations in Mizoram (SHALOM) helped in gaining access to different community groups.

### 2.2. Data collection tool

Development of IDI guides and probes was informed by a rapid review of HIV situation in Mizoram (16) conducted by us, These tools were further refined through discussions among the research team members some of whom were from Mizoram. The following domains were identified; ‘HIV situation in Mizoram’, ‘vulnerability to HIV’, ‘availability of health care services and access to them’, ‘roles of organizations’, ‘government supported targeted interventions (TI)’, ‘HIV prevention’ and ‘ways suggested for improvement’. Guides and probes were developed in English and later translated to Mizo language. The field staff were trained through online sessions on administration of guides and probes during interviews. These tools, were pilot tested and further modified for better comprehension and appropriateness.

### 2.3. Data collection

During October through December, 2020, two project technical officers (PTOs) from Mizoram; one male and one female (native speakers), with experience in qualitative research, along with two public health consultants (one male and one female) conducted key informant and in-depth-interviews with the participants till data saturation was reached (17).

### 2.4. Study settings

While public health consultants joined through virtual platforms due to COVID-19 related inter-state travel restrictions, the PTOs and field investigators were present physically in field settings. Interviews were conducted as per convenience and at the privacy of the participants. The PTOs and field investigators were able to physically travel to 4 study districts (Aizawl, Mamit, Lunglei and Kolasib) to facilitate interviewing through hybrid mode. Interviews in Champhai had to be conducted entirely online by the team as lockdown in Mizoram was made stringent due to the rising number of COVID-19 cases. The participant information sheet and informed consent form were administered to each participant (adults only) in a language that they could understand and their questions and clarifications, if any, were addressed. Once each participant signed a written informed consent form and provided permission for audio-recording, in-depth-interviews (IDI), were conducted maintaining COVID-appropriate behavior (Figure 1 presents district-map with respondent matrix). Interviews were conducted either in English or Mizo depending on the comfort and comprehension ability of the participants. Each interview lasted between 45 and 60 minutes following which field notes were created to capture observations and reflections of the moderators and note-takers.

### 2.5. Data analysis

Digitally recorded interviews were transcribed *ad verbatim* and interviews conducted in Mizo-language were later translated to English; pseudonyms, group or organizational affiliation were used as attribution code. Transcriptions and translations were carried out in collaboration with the Department of Social Work, Mizoram University. Quality checks of the translations were conducted on a regular basis, which were then used for coding and development of code-books. Interviews were read and re-read along with the field notes and debriefing notes and codes were developed through consensus among team members. Differences in opinion about coding among research team members were resolved through team meetings with research supervisor. We used N-vivo (version 11, QSR International) for organizing qualitative responses. Field notes prepared by the investigation team members before and after interactions with the study participants helped in gaining reflexivity. Data triangulation was carried out based on perspectives obtained from the participants with various lived experiences and reflections recorded in field notes.

## 3. Results

**Figure 2:**
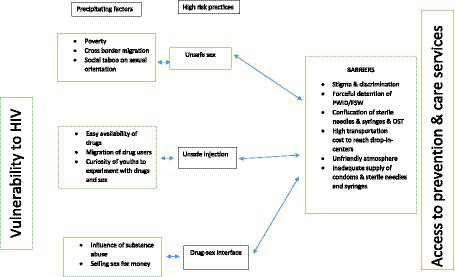
Analytical framework.

The reasons for the rise of HIV in Mizoram, population level vulnerability and barriers and facilitators around HIV prevention and care services were highlighted by the participants during IDIs. We captured them in an overall analytical framework (Figure 2) showing linkages between unsafe sex, early initiation of injection drug use, drug-sex interface, and social taboo against same-sex sex. Moreover, the framework presents some of the barriers; environment being non-friendly and stigmatizing, stock outs of supplies and fear of getting recognized at the service centers etc. The content analysis of the interviews identified three thematic layers ‘vulnerability to HIV,’ ‘prevention and care services,’ and ‘program elements and future roadmaps,’ which are presented below.

### 3.1. Vulnerability to HIV

In this study, it was highlighted across different population groups that people, who were aware of their HIV sero-reactive status, did not necessarily reveal it to their partners thereby lacking treatment supporters from their own families and also contributing to further spread of HIV. Different districts witnessed similar concerns surfacing.

*“Aizawl [is] number one because we know their way of living in which sex is high and substance use as well, and in Champhai there are lots of people using No 4 [local term for heroin] injection … similarly Khawzawl, which is close to Champhai … has many substance users that are in TNT (Thutak Nunpuitu Team) [Thutak Nunpuitu Team (TNT) undertakes rehabilitation for the depressed and maintains a De-addiction Centre] Champhai … so, I think for Khawlzawl risk is high”*

*- MZP male member, Champhai*

#### 3.1.1. Youths

Most of the respondents cited youths in the age group 21–34 year being most vulnerable to HIV. Students, as young as those in eighth standard in schools, reportedly had injected drugs. Respondents from different districts identified ‘easy availability of drugs,’ ‘peer pressure,’ and ‘curiosity to experiment’ as some of the factors contributing to injecting practices. Most of the PWID reportedly hailed from broken families.

*“There are so many things to say … . at young age I started doing drugs at the age of 16”*

*-PWID client under TI, male, Mamit*

#### 3.1.2. MSM

A few participants reported incidents of forced sex in district jails, at rehabilitation homes and house parties organized by MSM themselves.

*“Among MSM, there are those who are queer … and then there are bisexual … married men, you know … these bisexuals, not saying that they are positive … but they have been around a lot, are hoping to get in touch with younger MSMs … there is no way of reaching out to these kind of people … and how to warn others”*

*-MSM not with TI, Kolasib*

#### 3.1.3. PWID and FSW: drug-sex interface

Indo-Myanmar border of the district of Champhai serves as an important business corridor and it is porous enough to allow entry of ‘number 4.’ Even though the drug peddlers were punished, the charges slapped against them was such that on many instances they could easily bail themselves out. An Integrated Counselling and Testing Centre (ICTC) counselor further reported that during the COVID-19 pandemic, the number of injecting drug users in the community increased due to lesser vigilance across borders. Drug-sex interface was highlighted as an additional layer of vulnerability contributing to the progression of HIV epidemic during in-depth-interviews across all five study districts.

*“It’s mainly the female injecting drug users here … female injecting drug users when they are suffering from withdrawal syndrome and are in dire need of injecting drugs, they are not hesitant to sleep with others … if they are going to acquire more drugs or more money to buy drugs, even though they are not female sex workers there are number of female injecting drug users who get involved in such acts.”*

*-PWID client under TI, male, Champhai*

*“Especially the young ones just don’t want it (registration with Targeted Intervention sites) … they are afraid that their identity will get known … but they secretly are active in sex work, they secretly do drugs.”*

*- FSW PE under TI, Aizawl*

Financial needs and broken families were identified by some of the respondents as factors forcing women into sex work. Alcohol use, in this context, was considered having a negative impact upon the ability to negotiate condom use during sex work and in-turn increasing HIV-vulnerability. It was further reported that some of the clients would negotiate with the FSWs to pay higher to have condomless sex.

*“yes … he was a non-Mizo … we had sex because we needed the money not because we wanted to, not because we enjoyed it … my tears rolled down, I did it with tears falling from my eyes … I was distressed, I couldn’t even look at his face, I didn’t want to, but we are so desperate for money, it left us no choice but to do”*

*-PLHIV, female, Lunglei*

*“The fact that Aizawl is a business center for many contract workers. … when these workers earn more money … they tend to splurge. Some hire local 'call girls' or prostitutes nicknamed 'KS' (short for Khopui service or city service)”*

*–MHIP female member, Aizawl*

A rare point of view, as follows, was shared about why some women entered into sex work.

“We suffocate when we are under a lot of discipline, so we feel free when we go out any way we want, we want our freedom.”

-FSW-TI client, Aizawl

Noticeably, Young Mizo Association (YMA) and church leaders, at times, played a key role in forceful detention of drug users in central and district jails. According to the central jail superintendent from Aizawl district, about a fifth of the prison inmates were living with HIV and were under HIV services.

The other cited reason was movement of people in and out of Mizoram for work or education and unprotected sex with multiple partners during such period away from home. Reportedly, in Mamit district, Chakmas and Brus (ethnic tribes predominant in Mamit apart from Mizos) were difficult to reach out to due to their migratory nature; low awareness around HIV in them was also of concern.

### 3.2. Prevention and care services

Most of the participants, across the study districts, were aware of the availability of HIV prevention and care services; however, majority of them indicated that people did not get registered at TI sites due to fear of their identity getting disclosed. Coercive and discriminatory measures taken by the local community leaders and Young Mizo Association (YMA), forcefully confiscating condoms and sterile needles and syringes, were highlighted. Harassment of the PWIDs not only by local Mizo community but also by the law and enforcement personnel was also underlined

*“We are facing such problems from the Law and Enforcement personnel … apart from harassment our rights are also violated because when they see an addict nearby, they would take them in for no reasons and force them into [free manual labor] … making them remove the bushes from the surroundings of their residential compound … making them clean their vehicles … after all these they would eventually let them go … instances and reports like this are very common here in Champhai”*

*-PWID male member from TI, Champhai*

*“It (making sterile needles and syringes available) is being criticized by YMA Leaders … they thought we were distributing syringes and supporting drug use … but it was exchange [new ones against old ones] … we did because it was to be done … it should be told and known that we are not doing it to support drug use but it is to prevent HIV”*

*-Official from DACO, male, Champhai*

**Figure 3:**
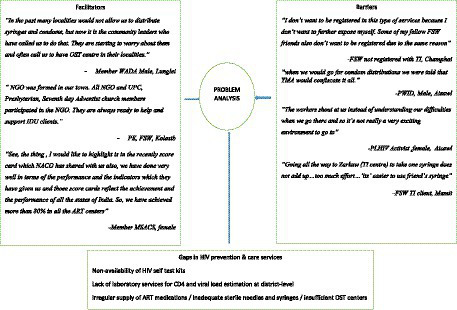
Barriers and facilitators: generalized HIV epidemic in Mizoram.

Recent examples of YMA and church leaders facilitating HIV awareness programs, and harm reduction services, were referred to as well. Some of the hurdles to access HIV prevention and care services were high transportation cost, fear of disclosure of identity, lack of services at flexible timings and long waiting period. Some of the respondents highlighted non-availability of ART medications and unpleasant behavior of staff as barriers to such services. Interrupted outreach activities due the COVID-19 related lockdown were other concern. Lack of MSM friendly TI sites at district-level was highlighted as one of the reasons for them not accessing health care facilities. Innovations such as HIV self-test were welcomed by most of the participants as they felt that they would be able to do it on their own and at their convenience. An abstraction matrix capturing the recorded barriers and facilitators for HIV prevention and care services is presented in Figure 3.

### 3.3. Program elements and suggestions for future roadmap

**Figure 4:**
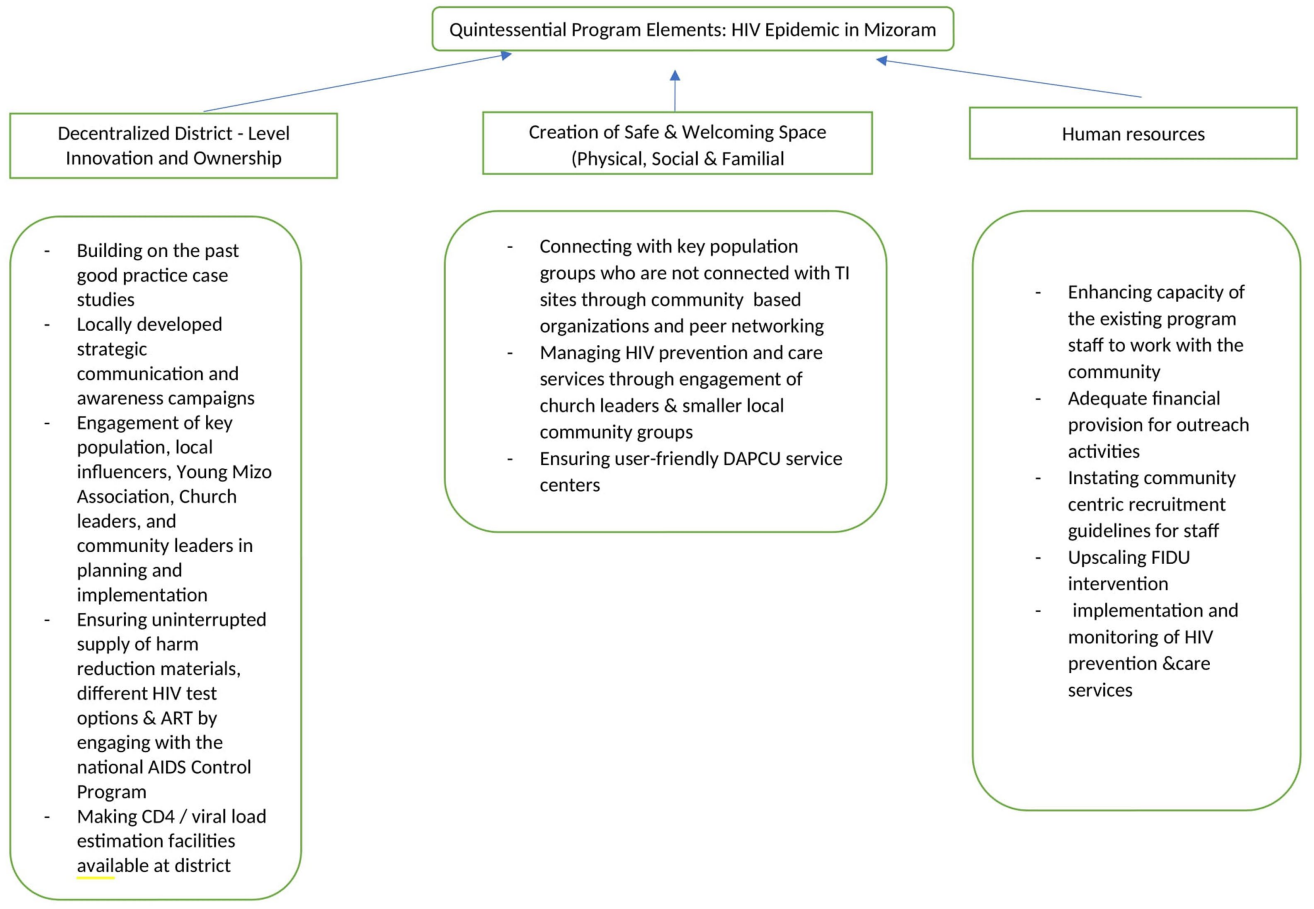
Future roadmap.

Respondents from different communities in districts reflected upon issues around ongoing HIV prevention and care services in Mizoram, which were pieced together to construct future roadmap presented in Figure 4. Some of the participants underlined the need for district specific measures such as engagement of Chakmas and Brus in community based interventions in the district of Mamit. The state program officials mentioned that the districts with a functioning District AIDS Program Control Unit (DAPCU) were performing better compared to the districts lacking them.

*“Collective coordination and effort in District and Local level, example Central YMA Level with State Government and MSACS for example we had big Control Society they need to be strengthened and other District Level Committee should be set up and should take steps”*

*-Male member, YMA*

*“In other District we can have whatever we plan but here in Aizawl and being headquarters we need the District Head and State Head, in rural it’s easy to have the Awareness program but not in Aizawl since it is the Capital, District Level Authority is not final, the decision is delayed.”*

*-Male member, DAPCU, Kolasib*

*“When they (patients) leave these ‘Homes’ (Rehabilitation homes) it is important that they are not forgotten and neglected too soon, but a follow up with an after-care program is imperative. Throwing them to jail or a Home is a short-term solution and is the same as putting them away for a while without any change in behavior”*

*-Male, Administrator, Incarceration facility*

## 4. Discussion

Uniqueness of this qualitative study rests with the exploration of perceptions of key population groups, youth leaders, service providers, policymakers and program officials on HIV/AIDS situation in high HIV burden districts of Mizoram. Importantly, this exploration was carried out by deploying some innovative interviewing means, as stringent restrictive measures due to the COVID-19 pandemic were in force. We were able to capture community voices and generate some valuable understanding around ‘how’ and ‘why’ of the rise of HIV epidemic in Mizoram through such processes. District specific aspects that contributed to such rise and ways forward to address them were identified as well where community engagement through restorative rather than retributive approach and decentralized local innovation appeared central.

Mizoram is one of the north-eastern states sharing international border with Bangladesh in the west and Myanmar in the east. It also has borders with other north-eastern States of India namely Tripura, Assam and Manipur. The porous nature of these borders is one of the reasons for easy availability of drugs at low cost (18) and illegal migration. In the current investigation, unsafe sexual practices and sharing of needles and syringes during drug use were cited as reasons for the upsurge of HIV epidemic in Mizoram. Adolescents reportedly got engaged in injecting drugs such as ‘number 4’ (heroin) at a very young age were associated with HIV-risk. It was also recorded that some of the female injecting drug users entered into sex-work to support their own drug use due to poverty. Thus, drug-sex interface between men and women surfaced as an intervention area requiring urgent attention. This was also revealed in a recent study from Mizoram (19). Young age were at onset of injecting drugs, as revealed by the present study and by other researchers, and during analysis of the National Integrated Bio-Behavioral Survey (IBBS) 2014–2015 (20, 21) and unsafe sexual practices, such as inconsistent condom use during casual and commercial sex, were individual-level risk behavior.

Despite fairly high level of HIV/AIDS awareness in Mizoram (22), study participants across the districts mentioned that among certain population groups, existed pockets of ignorance. There is a backlash from the Church leaders and Young Mizo Association toward the participants seeking HIV prevention and care services. Therefore, locally developed strategic communication and involvement of local influencers including Church and YMA could help in plugging such gaps and catalyze positive behavior change through restorative community building approach. Access of HIV prevention and care services by the key population groups and general mass could gain momentum through such community engagement and creation of safe and welcoming physical, social and familial space. HIV prevention and care activities thus need to be paired up with creation of a non-punitive environment so that the harm reduction interventions can reach out to the unreached. Noticeably, successful awareness activities were conducted in the past by the United Nations Office on Drugs and Crime (UNODC) by engaging red ribbon clubs and church leaders focusing on HIV test uptake by the youths (23). Church leaders and general population were sensitized through ‘Friends on Friday’ at Grace Hospital, Aizawl in mid-2000. The results of these initiatives were encouraging and could serve as ‘good practice’ (24).

As in the present inquiry, earlier investigations revealed that the stigma and discrimination experienced not only by the key population groups, but also general population, posed hindrance to access HIV prevention and care services. Rights based approach and active engagement of community stakeholders thus appeared crucial in this context to overcome such barriers (25, 26). Noticeably, engagement of religious leaders in HIV prevention led to encouraging results in improving HIV prevention and care services in Indonesia, Malaysia, and Kenya (27–29). Lessons learnt from these practices can be drawn upon as relevant for Mizoram.

The other issues of concern were stock-outs of condoms, occasional scarcity of ART medications and HIV testing kits, and scant availability of sterile needles and syringes at the right place and at flexible time and also unavailability of outreach workers and peer educators. An earlier study from Champhai highlighted similar issues on non-availability of sterile needles and syringes and stigma faced by female injecting drug users (FIDU) leading to delayed access to these services (30). The SACS and DAPCU need to take cognizance of such district specific issues and ensure uninterrupted HIV harm reduction services (31) by coordinating with the National AIDS Control Program, building upon active participation of people (32). Resulting community collectivization and empowerment could play a key role in attaining health and the desired goal of disease elimination (33).

Participants, in this study, highlighted the need for creating an enabling environment aligned with the local socio-cultural milieu and prevailing HIV situation. Ensuring active involvement of the community members in such interventions and implementing community centric recruitment guidelines for the staff would therefore be critical. Furthermore, strengthening the DAPCUs at district level will facilitate easy access to HIV testing and treatment services. Hyper-policing around harm reduction services such as needle syringe exchange program or social marketing of condoms yet remain a concern in the state of Mizoram and underlines the needs for sensitizing health care professionals and community influencers, through strategic communication.

All these macro-social and structural interventions would help attaining programmatic resilience during the time of stress such as COVID-19 pandemic. It is important to recognize at this point that the non-COVID health care services were negatively affected across the continents (Asia, Latin America and the Caribbean, Europe and Africa) during the pandemic (34). A study within India recorded that although the annual HIV testing was sustained among the vulnerable population groups such as female sex workers, men having sex with men, transgender population and truckers, it was not so for migrants and PWID (35).

We conclude that changing HIV intervention approaches, which have not been successful over a few decades despite repeated attempts, and reflecting upon ‘how to effectively engage community’ would require listening to the community voices. Creating an environment that would allow breaking the barriers built through stigma, discrimination and socio-economic differences also appears critical. HIV prevention and care services can thus be galvanized in the State of Mizoram with a sense of urgency and in a manner that they reach out to the unreached with the help of macro-social and structural support. Community’s involvement as agent of health and subjective wellbeing of the groups vulnerable to HIV should remain central in such endeavors along with other innovative program elements based on renewed understanding developed through the present qualitative inquiry.

## Data availability statement

The raw data supporting the conclusions of this article will be made available by the authors, without undue reservation.

## Ethics statement

The studies involving human participants were reviewed and approved by Institutional Ethics Committee ICMR-National AIDS Research Institute, Pune (NARI/EC Approval/20-21/409 dated 19th August 2020). The patients/participants provided their written informed consent to participate in this study.

## Author contributions

AR: monitoring, supervision of the study, data analysis and interpretation, quality check, and supervision and drafting of the manuscript. MM: monitoring, supervision of the study, data analysis and interpretation, quality check, and supervision and drafting of the manuscript. SP: conceptualization, design, methodology, research supervision, and finalizing the manuscript. HP: field implementation, data coordination, quality check and supervision of transcription and translation. HV: conducting interviews, preparing field notes, quality check. L: conducting interviews, preparing field notes, and quality check. TR: conducting interviews, preparing field notes, data analysis, and quality check. NL: conducting interviews, preparing field notes, data analysis, and quality check. All authors contributed to the article and approved the submitted version.

## Funding

The project received funds from Joint United Nations Program on HIV/AIDS (UNAIDS) (PR number 2020/1023250).

## Conflict of interest

The authors declare that the research was conducted in the absence of any commercial or financial relationships that could be construed as a potential conflict of interest.

## Publisher’s note

All claims expressed in this article are solely those of the authors and do not necessarily represent those of their affiliated organizations, or those of the publisher, the editors and the reviewers. Any product that may be evaluated in this article, or claim that may be made by its manufacturer, is not guaranteed or endorsed by the publisher.
